# Implementing a multifaceted tailored intervention to improve nutrition adequacy in critically ill patients: results of a multicenter feasibility study

**DOI:** 10.1186/cc13867

**Published:** 2014-05-11

**Authors:** Naomi E Cahill, Lauren Murch, Deborah Cook, Daren K Heyland

**Affiliations:** 1 Department of Public Health Sciences, Queen’s University, 99 University Ave, Kingston, ON K7L 3N6, Canada; 2 Clinical Evaluation Research Unit, Angada 4, Kingston General Hospital, 76 Stuart Street, Kingston, ON K7L 2 V7, Canada; 3 Departments of Medicine, Clinical Epidemiology & Biostatistics, McMaster University, 1280 Main St W, Hamilton, ON L8S 4L8, Canada; 4 Departments of Medicine, Queen’s University, 99 University Ave, Kingston, ON K7L 3N6, Canada

## Abstract

**Introduction:**

Tailoring interventions to address identified barriers to change may be an effective strategy to implement guidelines and improve practice. However, there is inadequate data to inform the optimal method or level of tailoring. Consequently, we conducted the PERFormance Enhancement of the Canadian nutrition guidelines by a Tailored Implementation Strategy (PERFECTIS) study to determine the feasibility of a multifaceted, interdisciplinary, tailored intervention aimed at improving adherence to critical care nutrition guidelines for the provision of enteral nutrition.

**Methods:**

A before-after study was conducted in seven ICUs from five hospitals in North America. During a 3-month pre-implementation phase, each ICU completed a nutrition practice audit to identify guideline-practice gaps and a barriers assessment to identify obstacles to practice change. During a one day meeting, the results of the audit and barriers assessment were reviewed and used to develop a site-specific tailored action plan. The tailored action plan was then implemented over a 12-month period that included bi-monthly progress meetings. Compliance with the tailored action plan was determined by the proportion of items in the action plan that was completely implemented. We examined acceptability of the intervention through staff responses to an evaluation questionnaire. In addition, the nutrition practice audit and barriers survey were repeated at the end of the implementation phase to determine changes in barriers and nutrition practices.

**Results:**

All five sites successfully completed all aspects of the study. However, their ability to fully implement all of their developed action plans varied from 14% to 75% compliance. Nurses, on average, rated the study-related activities and resources as ‘somewhat useful’ and a third of respondents ‘agreed’ or ‘strongly agreed’ that their nutrition practice had changed as a result of the intervention. We observed a statistically significant 10% (Site range -4.3% to -26.0%) decrease in overall barriers score, and a non-significant 6% (Site range -1.5% to 17.9%) and 4% (-8.3% to 18.2%) increase in the adequacy of total nutrition from calories and protein, respectively.

**Conclusions:**

The multifaceted tailored intervention appears to be feasible but further refinement is warranted prior to testing the effectiveness of the approach on a larger scale.

**Trial registration:**

ClinicalTrials.gov NCT01168128. Registered 21 July 2010.

## Introduction

Clinical practice guidelines on nutrition therapy in the ICU have been published to help clinicians make decisions regarding feeding their critically ill patients [1-5]. Although there are several discrepancies between guidelines on other topics, there is agreement for recommendations pertaining to enteral nutrition (EN) [6]. Energy and protein targets are more likely to be met if these guideline recommendations are followed [7]. However, numerous reports highlight that the quality of nutrition care is poor [8-12], with ICUs providing less than 60% of prescribed calories and protein [8]. Efforts to close this gap between guideline recommendations and actual practice are warranted [13].

There have been three cluster randomized controlled trials (RCTs) employing multifaceted educational interventions to implement nutrition guideline recommendations [14-16]. These RCTs observed small improvements in nutritional outcomes but no impact on clinical outcomes. Since then, the importance of adapting guidelines to the local context and identifying barriers to change has been recognized [17]. In the complex high-technology environment of the ICU, multiple factors can hinder the provision of adequate EN. Tailoring intervention strategies to take account of these barriers may thus result in greater improvements in nutrition practices compared with nontailored guideline implementation efforts [18]. A Cochrane review identified 26 RCTs that adopted this tailored approach to guideline implementation [18]. Most of these trials were conducted in a primary care setting, targeting physician prescribing behavior. While the impact on process outcomes varied both across and within studies, it appears that interventions tailored to overcome identified barriers are more effective at changing practice than no intervention or passive dissemination of guidelines. However, the optimal methods of identifying barriers and selecting interventions to address these barriers are unclear.

Given the complexity of the proposed tailored approach, prior to formally evaluating its impact on nutrition practice in a large representative sample of ICUs, it is prudent to first complete preliminary work [19]. To this end, we conducted a multiple case study to qualitatively explore the factors influencing adherence to critical care nutrition guidelines [20] and proposed a framework for categorizing the identified barriers [21]. We subsequently developed and validated a questionnaire to assess barriers to the provision of EN (see Additional file 1) [22]. Finally, we conducted the PERFormance Enhancement of the Canadian nutrition guidelines by a Tailored Implementation Strategy (PERFECTIS) study [ClinicalTrials.gov: NCT01168128] to evaluate whether a site-specific tailored plan was feasible in the critical care setting, and to generate preliminary evidence of its impact on ICU nutrition performance.

## Materials and methods

### Study design and overview

**Figure 1:**
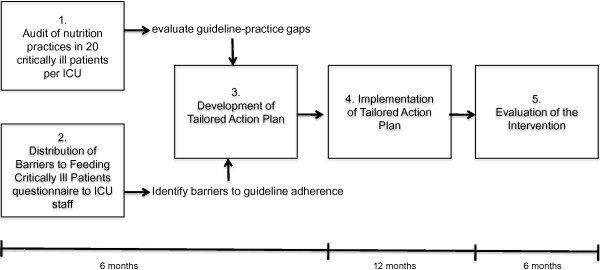
**Study schema.** The tailored action plan was developed through a five-step process: step 1, nutrition practice audit to determine gaps between guideline recommendations and actual practice; step 2, staff survey to identify barriers to enterally feeding patients; step 3, focus group to prioritize these barriers and brainstorm interventions to overcome the prioritized barriers; step 4, a 12-month implementation phase including bimonthly progress meetings; and step 5, evaluation of the intervention.

We conducted a before–after study to evaluate the feasibility of a tailored intervention to improve the provision of EN in the ICU (Figure 1 shows the study schema and Table 1 presents the study questions and evaluation criteria). To maximize the potential that participating sites would benefit from the intervention, ICUs had to meet the following inclusion criteria: a minimum of eight beds (smaller units do not routinely care for patients ventilated for >24 hours and who therefore require EN); affiliated with a registered dietitian (a predictor of higher nutrition performance [23]); located in North America (EN guideline recommendations in Canada and the USA are similar [1,3,5]); previous nutrition audit demonstrating average nutrition adequacy was <60% [23] (our goal was to improve nutrition practice, and lower baseline performance has been associated with greater improvement [24]); and demonstrated ability to collect the required data (that is, entered complete data on 20 patients in previous nutrition audit [23]). In addition, we purposefully aimed to include a mix of teaching status (teaching vs. nonteaching) and ICU types (open vs. closed) because these factors can influence nutrition practice [20,25].

**Table 1:** Study questions, evaluation criteria and outcomes for evaluating the feasibility of the tailored intervention

**Study question**	**Evaluation criteria**	**Achieved**				
		**Site 1**	**Site 2**	**Site 3**	**Site 4**	**Site 5**
1. Are we able to engage ICU staff to participate in the study?	Creation of a local guideline implementation team composed of at least one dietitian, one physician, and one nurse	√	√	√	X	√
	• Achieve a minimum of 35 responses or an overall response rate of 50% to the barriers questionnaire	√	X	√	X	√
	• Attendance of local guideline implementation team and key stakeholders at a 1-day meeting to develop the tailored intervention	√	√	√	√	√
2. Are sites able to prioritize barriers and select interventions to overcome these barriers?	Conduct of 1-day tailored action plan development meeting	√	√	√	√	√
	• Tailored action plan documented including step-by-step plan for implementation	√	√	√	√	√
3. Are sites able to implement the developed tailored intervention?	• Local guideline implementation team attendance at bimonthly progress teleconferences	√	√	√	√	√
	• Compliance with the tailored action plan	See Table 4				

Participating ICUs were recruited through an international ICU network for quality improvement [23]. Of the 179 ICUs, 14 sites met the inclusion criteria (that is, 76 sites were excluded because they did not succeed in entering data on 20 patients, 21 sites were excluded because they did not have a feeding protocol in place, six sites were excluded because they did not have a dietitian, and 62 sites were excluded because they were not located in North America and/or they achieved >60% prescribed calories received in a previous nutrition practice audit). An invite to participate in the PERFECTIS study was sent to all 14 eligible ICUs, and seven of these accepted. Three of these ICUs were geographically separate units in one hospital, but because of common infrastructure and staffing they developed and implemented one tailored action plan for all three units. Reasons for nonparticipation included the contact person no longer working in the ICU, lack of infrastructure to support research, inadequate time to dedicate to the study, and competing research studies. Characteristics of participating ICUs are presented in Table 2, reflecting a range of sizes, closed and open structures, teaching and nonteaching institutions, and two healthcare systems.

**Table 2:** Characteristics of participating ICUs

**Site**	**Country**	**Hospital type**	**Hospital size (beds)**	**ICU structure**^**a**^	**ICU size (beds)**	**Medical director**	**Clinical specialty**	**FTE dietician per 10 beds**
1	USA	Nonteaching	315	Closed	20	Yes	Mixed medical/surgical	0.2
2	Canada	Teaching	587	Closed	16	Yes	Mixed medical/surgical	0.4
3a	USA	Teaching	600	Open	12	Yes	Surgical trauma	0.4
3b	USA	Teaching	600	Open	10	Yes	Neurological	0.5
3c	USA	Teaching	600	Open	10	Yes	Medical	0.5
4	Canada	Nonteaching	420	Open	13	Yes	Mixed medical/surgical	0.5
5	Canada	Teaching	830	Closed	30	Yes	Mixed medical/surgical	0.4

Characteristics based on 2011 data collection. FTE, full-time equivalent. ^a^Closed, under the care of an intensivist; open, under the care of any attending physician.

An interdisciplinary local guideline implementation team consisting of the ICU dietitian(s), attending physician, and a nurse was formed at each site. Team members self-identified as local nutrition opinion leaders. The local teams were responsible for study coordination, data collection, and implementing the tailored intervention.

### Intervention

The overall design of the intervention was informed by Graham and colleagues’ knowledge-to-action model, which describes the necessary steps for implementation of knowledge [26]. The barriers assessment was guided by our previously developed framework for understanding barriers to critical care nutrition guideline recommendations [21], and the approach to addressing identified barriers (that is, tailoring) was informed by the Barriers Identification and Mitigation Tool developed by Gurses and colleagues [27]. In addition, in designing the intervention we were cognizant of the feedback from participants of a previous cluster RCT evaluating nutrition guideline implementation conducted by our research group [15] and existing literature on tailoring interventions to overcome barriers [17]. The components of the intervention are described in Table 3. Several of the change strategies were common across participating sites (that is, audit and feedback, educational outreach, performance coaching, opinion leaders, networking meeting).

**Table 3:** Description of intervention

**Intervention**	**Description**	**Rationale**	**Example of activity/resource**
Audit and feedback	Summary of nutrition performance data collected by abstracting data from the charts of 20 consecutive mechanically ventilated critically ill patients	Demonstrating the gap between actual and desired performance motivates providers to change practice to reduce the gap	Benchmarked performance report comparing current nutrition practice with guideline recommendations and with other ICUs
			Review of performance with small group, discussion of reasons for poor performance, and identification of opportunities for improvement
Educational outreach visit	Personal visit by an external nutrition expert to critical care providers in their own setting, including:	Current evidence-based information is communicated to providers, increasing their knowledge of nutrition, awareness of guideline–practice gaps, and leading to practice change	Grand rounds with ICU providers
			Face-to-face discussions with physicians
	1. a 1-hour interactive presentation with the following content:		
	• evidence supporting nutrition guideline recommendations		
	• strategies to optimize EN		
	• rationale for tailored intervention		
	2. feedback on nutrition performance		
	3. opportunity for discussion		
Tailored action plan to overcome identified barriers	Site-specific bundle of interventions selected to overcome local barriers to the provision of EN. Developed at 1-day meeting attended by the local guideline implementation team and key stakeholders and facilitated by the external research team; involving identification of and prioritization of barriers to target for change, brainstorming of feasible and impactful solutions, and development of a step-by-step action plan for implementation. Action plan included interventions targeting at both individual provider and system supports	Strategies selected to address identified barriers, reduce the influence of these barriers leading to practice improvements	System/organizational:
			• addition of EN initiation to ICU admission order set
			• stock of enteral formula in the ICU
			Individual provider:
			• education through noon hour workshops (that is, ‘lunch and learns’)/bedside huddles (that is, brief small group meetings held on the unit)
			• information sheets summarizing current evidence/guideline recommendations
			Reminders:
			• posters
			• checklist
Performance coaching	External research team provide support to the local guideline implementation team while they implement their action plan	By receiving advice and guidance while going through the action plan implementation process, local teams are more likely to achieve their goals	Facilitation of bimonthly teleconference calls monitoring the progress of the implementation of the tailored action plans
Local opinion leaders	Physician, dietician, and nurse who work in the ICU and are knowledgeable about nutrition therapy	Opinion leaders change practice by influencing the attitudes and behavior of their peers through informal guidance	Informal discussions at the bedside regarding provision of EN to the patients
Networking meeting	Half-day meeting with all participating sites, where each site present the successes and challenges experienced implementing their action plans	Engaging with others with similar experiences leads to sharing of knowledge and motivates change	Informal discussions

EN, enteral nutrition.

The main component of the intervention was the development and implementation of an action plan tailored to local barriers. These plans aimed to address both individual and organizational barriers amenable to change rather than nonmodifiable barriers (for example, hospital teaching status and case mix). The development and implementation of these site-specific tailored action plans have been described elsewhere and are summarized in Figure 1[28]. In brief, following an audit of nutrition practices to identify guideline–practice gaps at each site and the distribution of the barriers to feeding critically ill patients questionnaire to all full-time and part-time ICU physicians, managers, dietitian(s), and nurses, the research team visited each site over a period of 1 month and facilitated face-to-face meetings with the local team and key stakeholders (for example, ICU manager, nurse manager, intensivists, dietitians, nurses, clinical educators) at each site. The number of attendees at the meetings ranged from three to 14 individuals across the five sites. These 1-day meetings were facilitated by the external research team (NEC, LM, and DKH), who guided the discussions by posing questions and prompting attendees to think about their nutrition practice, but they did not make suggestions regarding the content of the action plan. The first half of the meeting was dedicated to reflecting on current nutrition practice, identifying and prioritizing barriers to the optimal provision of EN. Through review of the results of the practice audit, attendees identified areas of nutrition practice to target for improvement, and explored reasons for poor performance. The results of the barriers questionnaire were also reviewed and the main barriers to enterally feeding patients were identified. Based on these data and the personal experiences of the key stakeholders, the barriers were ranked in order of perceived impact on nutrition practice.

Once the barriers had been prioritized, the second half of the meeting involved brainstorming strategies to overcome the barriers. The selection of strategies was based on consideration of the feasibility of implementing the proposed change and the impact that this change would have on the provision of EN. For each selected strategy or action item, attendees developed a step-by-step plan for implementation that included the what, who and when for each step and how successful implementation would be assessed. Where applicable, ICUs were encouraged to adapt the guideline recommendations to their local context (for example, incorporation into bedside algorithms, Computer Patient Order Entry systems, local policy documents, and so forth). In addition, the research team developed templates of various educational materials and bedside tools that were available for adaptation by the sites.

The study took place between September 2009 and September 2011, and the tailored action plans were implemented over 12 months (May/June 2010 to May 2011). During the implementation phase, bimonthly teleconferences were held between the local guideline implementation team and the research team to monitor progress.

### Data collection and management

Data on nutrition practices were collected as part of the ongoing International Nutrition Survey [8,23]. Data collection details were reported previously [8]. Starting on 16 September 2009 and 11 May 2011, the local guideline implementation team at participating ICUs identified 20 consecutive adult patients who were mechanically ventilated within the first 48 hours of ICU admission and who remained in the ICU for more than 72 hours. Data were retrospectively abstracted from hospital records on patient characteristics and baseline nutrition assessment (that is, body mass index, methods used to calculate nutritional requirements, energy and protein prescribed by the dietitian). Daily nutrition information was collected on the type (route of delivery, type of solution provided) and amount (total calories and protein received) of nutrition, as well as strategies to enhance delivery (motility agents and small bowel feeding tubes) and morning blood glucose. Daily information was recorded from ICU admission for a maximum of 12 days unless death or ICU discharge occurred sooner. Data on head of the bed elevation were obtained through direct observation on the day of enrollment. Patients were followed up to determine their ICU and hospital outcomes at 60 days. Data were entered using a secure web-based data collection tool (REDCap Software, Version 3.3.0, © 2012 Vanderbilt University, http://project-redcap.org/).

In March/April 2010 and May/June 2011, the barriers to enterally feeding critically ill patients questionnaire was administered to all full-time and part-time ICU physicians, managers, dietitian(s), and nurses (see Additional file 1). If more than 85 nurses were employed, a sample of 60 nurses was identified at each site by simple random sampling without replacement. The barriers to feeding critically ill patients questionnaire was developed for this study [29]. Based on feedback following baseline administration, the questionnaire was revised. In this report we focus on items that were common to both versions of the questionnaire, namely a list of 21 potential barriers to delivery of EN divided into five subscales: guideline recommendations and implementation; ICU resources; dietitian support; delivery of EN to the patient; and critical care provider attitudes and behavior. Respondents were asked to rate on a seven-point Likert scale the importance of each item as a barrier in their ICU. To maximize the response rate, the questionnaire was distributed according to a modified Dillman’s tailored design method [30], including a precontact memo, multiple reminders, and sending a second copy of the questionnaire. The modes of distribution and capturing responses (that is, web vs. paper based) were determined by the local guideline implementation team. The questionnaires were either emailed, hand delivered, or placed in staff mailboxes.

To determine compliance with the tailored action plan, at the end of the 12-month implementation phase the local guideline implementation team ranked their progress towards implementing each action using the Institutes for Healthcare Improvement Assessment Scale for Collaboratives [31], a scale where 0 = no action, 1 = initial steps taken but no steps complete, 2 = implementation in progress and some steps complete, 3 = implementation 50% complete, 4 = implementation 100% complete, and 5 = target/objectives exceeded. To further evaluate the intervention, in May/June 2011 a brief questionnaire was distributed to ICU staff using the same methodology as for the barriers questionnaire. Respondents were asked about their exposure to and usefulness of each action in their tailored action plan using a scale where 1 = useless and 5 = very useful. In addition, we asked about nutrition practice change as a result of PERFECTIS study participation.

### Outcome measures

Table 1 outlines our study questions and corresponding evaluation criteria for determining the feasibility of the tailored intervention. Compliance with the tailored action plan was defined as the proportion of strategies with a progress rank of 4 or 5 out of the total number of strategies in the site’s action plan. To further examine compliance with the intervention, we examined staff responses to the evaluation questionnaire.

To generate preliminary evidence to support the effectiveness of the tailored intervention we also evaluated change in barriers score(s) and change in nutrition practice indicators. Barriers scores were calculated by awarding 1, 2, or 3 points if the respondent identified an item as a ‘5 = somewhat important’, ‘6 = important’ or ‘7 = very important’ barrier respectively. If an item was rated 1 to 4 (that is, ‘not at all important’ to ‘neither important or unimportant’) it was awarded 0 points. The barriers score was calculated by dividing the awarded points for each item by the maximum potential points (that is, 3) and multiplied by 100. The overall, subscale and prioritized barriers score was the mean score awarded by respondents for all of the items, subscale items, and items selected as a priority for action by each site, respectively. Change in barriers scores was calculated as the score at baseline subtracted from the score at follow-up, with a decrease in score indicating a decrease in the perceived importance of the item.

Nutrition practice indicators evaluated included adequacy of calories and protein from EN, adequacy of calories and protein from total nutrition, proportion of patients who achieved >80% adequacy of calories from total nutrition within 72 hours of ICU admission, proportion of patients receiving EN, proportion of patients with EN initiated within 48 hours, time from start of EN to >80% adequacy of calories from total nutrition, proportion of patients with high gastric residual volumes receiving motility agents and/or small bowel tubes, mean head of bed elevation, and proportion of patients with hyperglycemia.

### Analysis

As the objective of this before–after study was to evaluate the feasibility of a tailored intervention to overcome barriers to adherence to ICU nutrition guideline recommendations, rather than to evaluate its impact on barriers score or nutrition performance, no formal sample size or power calculation was completed. Consequently, analysis of secondary outcome measures is hypothesis generating.

The purpose of the intervention was to address modifiable barriers; however, following tailored action plan development, each site identified items that were nonactionable or outside the locus of control of the local team (for example, purchasing additional feeding pumps, funding for additional dietitian time). Consequently, we calculated compliance for the original action plan (that is, primary analysis) and compliance omitting these nonactionable items (that is, secondary analysis).

The tailored intervention targeted change at the ICU level; therefore, all patient and provider-level data were aggregated to the site level and treated as site-level variables. Categorical variables are reported as counts and percentages and compared between baseline and follow-up by Fisher’s exact test. Continuous variables are described by their means and standard deviations or medians and interquartile range (IQR) and were compared using a linear regression model with mixed effects, permitting random intercepts to account for clustering within ICUs and ICU by year.

Total nutrition adequacy was calculated as the amount of calories or protein received (from either EN or appropriate parenteral nutrition (PN) – that is, presence of clinical contraindication to EN (namely, mechanical bowel obstruction, bowel ischemia, small bowel ileus, small bowel fistulae, gastrointestinal perforation, and short gut syndrome) but not oral intake – plus propofol divided by the amount prescribed as per the baseline assessment and expressed as a percentage. Days without EN or PN and days with inappropriate PN were included and counted as 0% adequacy. EN adequacy was calculated as the mean calories received from EN divided by the maximum amount prescribed as per the baseline assessment, expressed as a percentage and averaged over the ICU stay. Patients with a contraindication to EN were excluded from the analysis. Days without EN or days with PN were included and counted as 0% adequacy. Days following permanent progression to exclusive oral intake were excluded from the calculation of total nutrition and EN adequacy. To account for the confounding effect of duration of nutrition exposure, the prescribed calories received by each patient were adjusted for evaluable nutrition days [32]. Statistical analyses were completed using SAS v9.1.3 (SAS Institute Inc., Cary, NC, USA). All tests were two-sided with *P* < 0.05 considered statistically significant.

Institutional ethics approval was obtained, and the need for informed patient and staff consent was waived by the Health Sciences Research Ethics Board at Queen’s University, Kingston, ON, Canada and the five participating hospitals (see Acknowledgements).

## Results

All five participating ICUs successfully completed data collection at baseline and follow-up, and developed and implemented a tailored action plan. Table 1 outlines our assessment of feasibility as determined by our *a priori* evaluation criteria.

We determined that we were able to engage ICU staff to participate in the study, and that they were competent at prioritizing barriers and developing a tailored action plan (Study Questions 1 and 2). All sites created a local guideline implementation team. However, at Site 4 the team was not multidisciplinary, being composed of two dietitians. Overall, the number of team members at each site ranged from two to 10 individuals. On average, 37 (site range 29 to 52) barrier questionnaires were completed by ICU staff at each site for a mean response rate of 46% (site range 37 to 65%). Two sites did not achieve a minimum of 35 responses or an overall response rate of 50% (that is, 32/85 (38%) at Site 2 and 29/73 (40%) at Site 4).

Table 4 presents the primary and secondary analyses of compliance with the action plans. Across the five sites the developed action plans consisted of either seven or eight action items, and each site identified one item that was nonmodifiable with a progress rank of 0, with the exception of Site 2 that identified two such items. The median progress rank was 4, indicating implementation was 100% complete. For the secondary evaluation, omitting nonmodifiable barriers, the ability of sites to successfully implement their action items varied from achieving a 4 or 5 progress rank for one of the six action items (17% compliance) at Site 3, to six out of seven action items (86% compliance) at Sites 1 and 5. However, at the time of follow-up data collection, several sites had partially implemented action items and their efforts to complete implementation were ongoing.

**Table 4:** Evaluation of tailored intervention

	**Overall**	**Site 1**	**Site 2**	**Site 3**	**Site 4**	**Site 5**
**Compliance with the tailored action plan**						
Primary analysis of compliance with action plan^a^	57%	6/8 (75%)	4/8 (50%)	1/7 (14%)	5/7 (71%)	6/8 (75%)
Secondary analysis of compliance with action plan^b^	68%	6/7 (86%)	4/6 (67%)	1/6 (17%)	5/6 (83%)	6/7 (87%)
Progress rank for items in the action plan^c^	4 (0 to 5)	4 (2 to 5)	3.5 (0 to 5)	3 (0 to 5)	4 (0 to 5)	4 (0 to 4)
**Nurses responses to evaluation questionnaire**						
Know all members of Guideline Implementation Team	66/82 (80%)	12/13 (92%)	16/23 (70%)	15/23 (65%)	17/17 (100%)	6/6 (100%)
Discussed nutrition with Guideline Implementation Team daily or weekly	50/81 (62%)	10/13 (77%)	6/22 (27%)	17/23 (74%)	13/17 (77%)	4/6 (67%)
Prescribed calories received/caloric debt reported on rounds often or all the time	42/81 (52%)	9/13 (69%)	6/23 (26%)	12/23 (52%)	12/16 (75%)	3/6 (50%)
Agree or strongly agree that nutrition practice changed as a result of PERFECTIS	25/79 (32%)	7/13 (54%)	2/21 (9.5%)	9/23 (39%)	4/16 (25%)	1/6 (17%)
Number of PERFECTIS activities/resources as part of the action plan^d^	8	9	9	7	7	9
PERFECTIS-related activities/resources exposed to	7 (0 to 9)	7 (5 to 9)	3 (0 to 9)	7 (1 to 7)	7 (2 to 7)	6.5 (2 to 8)
Rating of usefulness of PERFECTIS activities/resources exposed to^e^	4 (1 to 5)	4 (1 to 5)	4 (1 to 5)	4 (1 to 5)	4 (1 to 5)	4.5 (2 to 5)

Data presented as percentage, median (range) or number (percentage). PERFECTIS, PERFormance Enhancement of the Canadian nutrition guidelines by a Tailored Implementation Strategy. ^a^The proportion of actions with a progress rank of 4 or 5 out of the total number of action items in the action plan. ^b^The proportion of actions with a progress rank of 4 or 5 out of the total number of action items in the action plan excluding items addressing nonmodifiable barriers with a progress rank of 0. ^c^Progress rank: 0 = no action, 1 = initial steps taken but no steps complete, 2 = implementation in progress and some steps complete, 3 = implementation 50% complete, 4 = implementation 100% complete, and 5 = target/objectives exceeded. ^d^Number of activities/resources may not correspond to the number of action plan items because some action items may have involved more than one strategy/resource (for example, development of protocol, education session, and newsletter article) and some strategies (for example, educational session) may have been employed for several action items. ^e^Rating scale: 1 = useless, 2 = somewhat useless, 3 = neutral, 4 = somewhat useful, 5 = very useful.

The questionnaire evaluating the implementation of the action plans was completed by 82 nurses (24% response rate). Eighty percent of respondents knew all members of the local guideline implementation team, and 59% had discussed nutrition with these members on a ‘daily’ or ‘weekly’ basis. As a result of the study, prescribed calories received or caloric deficit was reported on daily rounds ‘often’ or ‘all the time’, according to 52% of respondents; 32% ‘agreed’ or ‘strongly agreed’ that they had changed their nutrition practice as a result of study participation. On average, nurses were exposed to seven (site range three to seven) study-related activities or resources, and on average rated these as 4 = somewhat useful (see Additional file 2). Table 4 describes the results of the evaluation questionnaire by site.

A total of 182 critical care staff (134 (74%) nurses, 25 (14%) physicians, 12 (7%) dietitians and 11 (6%) other) responded to the barriers to enterally feeding critically ill patients questionnaire at baseline, and 118 (93 (79%) nurses, 12 (10%) physicians, 10 (9%) dietitians and three (3%) other) at follow-up; for an overall response rate of 45% (39% for nurses, 44% for physicians, and 100% for dietitians) and of 29% (27% for nurses, 21% for physicians, and 83% for dietitians) at the two respective time points. Respondent characteristics were similar at baseline and follow-up. Over one-half were experienced staff working in the ICU for >5 years, and two-thirds worked full-time.

**Figure 2:**
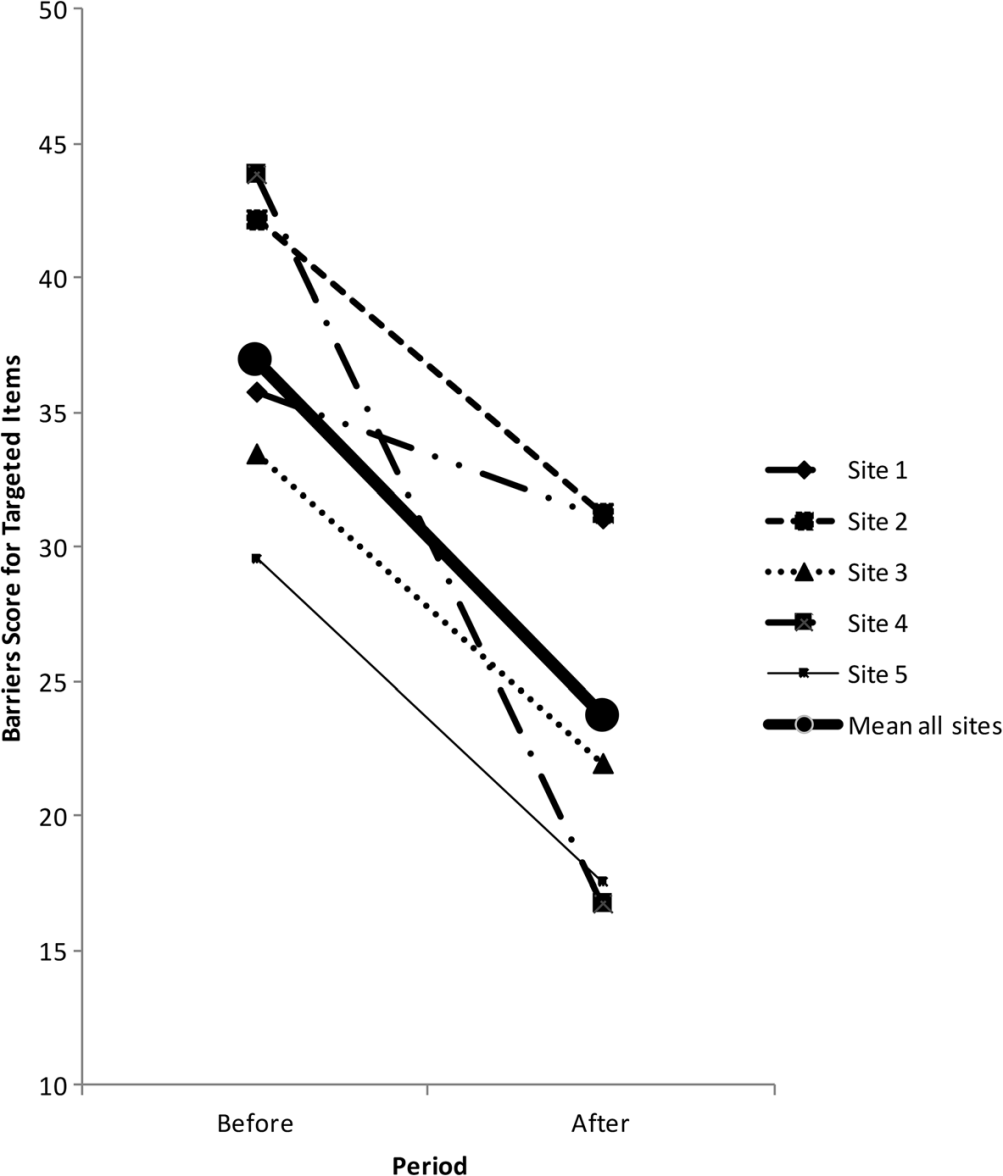
Change in prioritized barriers score for questionnaire items targeted by the tailored intervention overall and by site.

Figure 2 illustrates the change in prioritized barriers score, reflecting barriers targeted for improvement by the tailored action plans at each site. The prioritized barriers score decreased in all sites between baseline and follow-up with a mean change of –13 points, ranging from –5 (standard deviation 29) at Site 1 to –26 (standard deviation 19) at Site 4. We observed a 10-point (site range –4 to –26) reduction in overall barriers score. The barriers score decreased for all 21 items in the questionnaire and this change was statistically significant for 16 items (item range –1 to –18). The greatest change was observed in Subscale 4 (delivery of EN to the patient) and Subscale 5 (provider attitudes and behavior), with a change in barriers score of –12 points (–2 to –36) and –11 points (–3 to –22 respectively). Although the barriers score decreased at all sites for most items, the magnitude of change varied (see Additional file 3).

There were 140 patients accrued in the nutrition practice audit at baseline and 138 patients at follow-up. Patient characteristics and clinical outcomes were similar at both time points, 55% were male with a median age of 61 years (IQR 51 to 72 years) and body mass index of 27 kg/m^2^ (IQR 23 to 32 kg/m^2^). The majority was medical patients (80%) and the median Acute Physiology and Chronic Health Evaluation II score was 22 (IQR 17 to 28). The median energy and protein prescribed by the dietitian was 1,745 kcal (IQR 1,541 to 1,891 kcal) and 96 g (IQR 76 to 109 g) respectively. In 79% of patients, these energy requirements were calculated using a weight-based formula ranging from 20 to 30 kcal/kg. The protein prescriptions were also calculated using a weight-based formula (median 1.2 g/kg (IQR 0.9 to 1.5 g/kg)). Median lengths of mechanical ventilation and ICU stay were 5 days (IQR 2 to10 days) and 8 days (IQR 5 to14 days) respectively, and 60-day hospital mortality was 25.5%.

**Figure 3:**
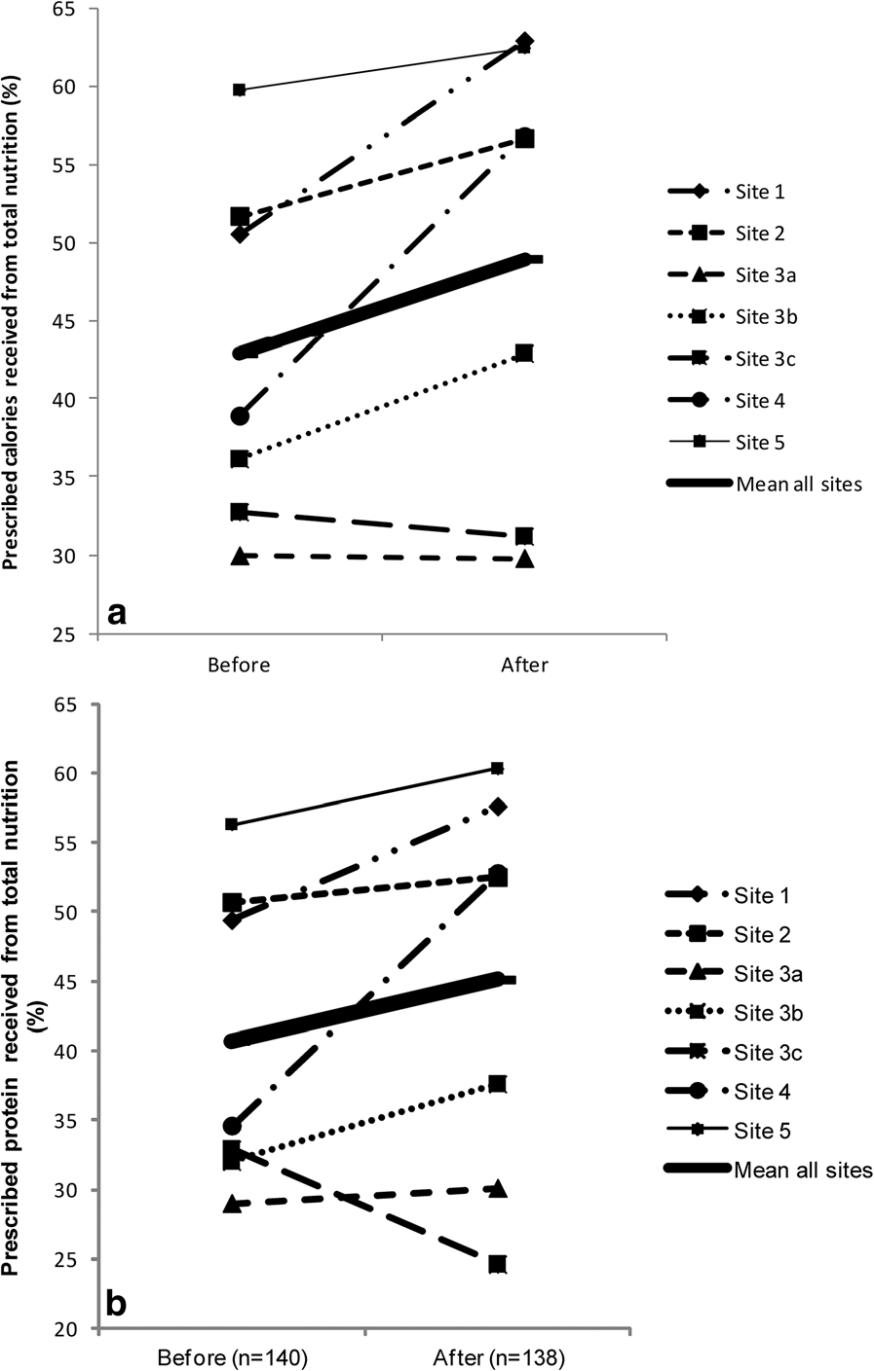
**Nutrition outcome measures. (a)** Change in adequacy of calories from total nutrition overall and by site. **(b)** Change in adequacy of protein from total nutrition overall and by site.

Figure 3a shows the change in caloric adequacy from total nutrition at each site. While some sites did not improve, an increase of >10% was observed at two sites (51 to 63% at Site 1, and 39 to 57% at Site 4). Similar results were observed for protein adequacy from total nutrition (Figure 3b and Table 5).

**Table 5:** Change in nutrition practice indicators

**Nutrition practice**	**Before (2009)**	**After (2011)**	**Change**	**Range**		***P *** **value**^**e**^
	**(*n*** **= 140)**	**(*n*** **= 138)**		**Min**	**Max**	
Adequacy of calories from total nutrition^a^ (%)	42.9 (29.6)	49.0 (31.2)	6.1	–1.6	18.0	0.23^f^
Adequacy of protein from total nutrition (%)	40.7 (31.6)	45.1 (31.8)	4.4	–8.3	18.2	0.67^f^
Adequacy of calories from EN (%)	36.1 (29.7)	37.6 (29.1)	1.4	–5.5	8.8	0.76^f^
	38.7 3(1.5)	40.3 (31.0)	1.6	–8.3	12.2	0.75^f^
Patients who achieved >80% adequacy from calories within 72 hours of ICU admission^c^	36 (26)	44 (32)	6	–15	30	0.45
Type of nutrition						
EN only	98 (70)	100 (72)	2	–12	15	
PN only	6 (4)	8 (6)	2	–5	5	
EN + PN	12 (9)	10 (7)	–2	–5	1	
None	24 (17)	20 (15)	–2	–15	12	
EN initiated within 48 hours^b^	71 (65)	77 (75)	10	–13	38	0.16
Time from ICU admission to initiation of EN (hours)^b^	40.3 (36.5)	39.8 (43.7)	–0.5	–25	23	0.94
Time from start of EN to >80% adequacy of calories (days)^c^	6.8 (3.8, 12)	5.8 (2.8,12)	–1.0	–7.6	1.1	
Use of motility agents in patients with GRV^d^	7 (50)	11 (58)	8	–50	2	0.88
Use of small bowel feeding in patients with GRV^d^	0 (0)	0 (0)	0	0	0	N/A
Head of bed elevation (degrees)	34.0 (17.2)	32.0 (5.8)	–2.0	–6.7	5.4	0.59
Morning blood glucose > 10 mmol/l (patient-days)	165 (16)	162 (15)	–1	–18	6	0.68^g^

Data presented as mean (standard deviation), *n* (%) or median (interquartile range). EN, enteral nutrition; GRV, gastric residual volume; Max, maximum; Min, minimum; N/A, strategy employed at single site only; PN, parenteral nutrition. ^a^Included propofol, EN, and appropriate PN. ^b^Only included patients who ever received EN. ^c^Based on data indicating that achieving >80% adequacy of calories is associated with decreased mortality [32]. ^d^Only included patients who ever had high GRV. ^e^*P* values account for ICU level clustering, using random ICU and ICU by year effects for continuous outcomes and the Rao–Scott chi-squared method clustering by ICU for categorical outcomes. ^f^Adjusted for evaluable nutrition days. ^g^*P* values account for ICU and patient level clustering using the Rao–Scott chi-squared method.

## Discussion

In this multicenter study of a tailored intervention to improve the provision of EN to critically ill patients, we demonstrated that this multifaceted, interdisciplinary intervention is feasible with all five sites successfully developing and implementing their action plans. However, the degree of implementation varied across sites, with no ICU completely implementing all proposed strategies in their action plan within the 12-month implementation phase. Although this study was not powered to evaluate differences in outcomes, we did observe significant decreases in barrier scores and small nonsignificant improvements in some nutrition practices.

These results contribute to the rapidly growing body of evidence on customized approaches to knowledge translation. The Cochrane review of tailored interventions published in 2010 identified 26 trials [18], 11 more than the 15 included in the 2005 publication [33]. Awareness of 14 ongoing studies on this topic for inclusion in the next update of this Cochrane review underscores how tailoring is being incorporated into guideline science. However, no prior or ongoing studies focused on nutrition guidelines or the ICU, raising questions about the generalizability of prior studies, and the need for context-specific evaluation. Our study provides new data on a tailored intervention in the acute care setting aiming to change a range of professional practices. The Cochrane review categorized the complexity and extent to which tailored interventions were adjusted to local barriers as low, moderate, or high. In our study, the complexity of both the barriers assessment and tailoring was ‘high’, meaning that we used multiple methods to identify site-specific barriers including a staff survey, provider focus groups, and nutrition performance data, customizing the intervention to site-specific barriers identified by local staff. A unique feature of our study was the development and implementation of a tailored action plan led by a local team rather than prescribed by external researchers, which proved feasible in teaching and nonteaching hospitals, open and closed ICUs, urban and rural locations, and in sites with demonstrated difficulties in adhering to nutrition guideline recommendations.

The effect of the tailored intervention was not uniform across sites. While the mean changes in nutrition indicators were not statistically or clinically significant, large changes were observed at some sites (that is, Sites 1 and 4); these sites were also the sites with the greatest reduction in barriers score, and highest compliance to the tailored action plan, thus supporting the assumption that the observed changes were due to our intervention. Consequently, to optimize practice improvements in all sites, we need a better understanding of the intra-institutional factors that either facilitated or hindered change at the site level. There may be unmodifiable barriers not targeted by our intervention that limit its potential effectiveness. Similarly, factors such as leadership support and ICU readiness to change may also preclude the ability to implement the action plan. Some of the observed variation may be due to differences in the change strategies employed by the sites or different degrees of uptake of action plan items. Given the nature of this multifaceted, complex intervention, we are unable to determine which elements of the intervention were effective or which were ineffective; further, we are unable to quantify the dose of each strategy that individual staff members received.

We also observed variation in the rate of implementation of the tailored action plans. The duration of the implementation phase was 12 months. While some sites only partially implemented their action plans in this time, others implemented each item within 6 months. In developing the action plans, sites were asked to consider the feasibility of completing each action within the study time frame. Understanding the reasons for the delays experienced by some sites and why some action items were not implemented may help future initiatives to set appropriate timelines or provide additional resources to support lagging sites. Our results suggest that sites may require more than 12 months to completely implement all of the planned changes.

The barriers to enterally feeding critically ill patients questionnaire was a survey instrument developed for this study [29]. Although we observed decreased barrier scores derived from the results of this questionnaire, indicating the staff perceived barriers to be less important following the tailored intervention, we are uncertain about the clinical significance of these change scores. To this end, to evaluate the construct validity of the barriers questionnaire we conducted a multilevel regression analysis with data from 55 ICUs from five geographic regions, and observed that a 10-point increase in overall barriers score is associated with a 5% decrease in total nutrition adequacy, thus providing some evidence to support that the barriers identified by this questionnaire are inversely associated with nutrition performance [34].

Our study has a number of limitations. First, the five ICUs were invited to participate from a group of ICUs previously participating in quality improvement initiatives. Observed practice changes may have been influenced by their prior involvement in quality improvement projects rather than the tailored intervention *per se*; furthermore, sites accepting the invitation to participate may differ from those declining, introducing selection bias. Second, the response rate to the barriers questionnaire was only 45% at baseline and 29% at follow-up, perhaps reflecting staff fatigue from frequent surveys external to this study or lack of interest in improving nutrition practice; consequently, a response bias may be operant if responding staff had a greater interest in nutrition than nonresponders. Third, compliance with the action plan was assessed by the local guideline implementation teams’ self-rating of progress; sites may therefore have rated their progress higher than the actual progress. However, when the action plans were developed, sites were asked to select objective criteria by which they could assess whether the action item had been implemented. These criteria guided the completion of the progress report. Fourth, we did not assess the cost-effectiveness of the intervention or the time commitment required by the local guideline implementation team. These are important factors to consider when assessing the feasibility of adopting a tailored approach. Finally, there are several components of our intervention that may limit its generalizability to the real world. The external research team played an active role in the intervention; presenting at grand rounds, facilitating the action plan development meetings, and coaching the local guideline implementation team through the implementation phase. Furthermore, all participating sites were affiliated with a registered dietitian who was a part of the guideline implementation team. ICUs without a dietitian or local nutrition expert may find it difficult to develop and implement a nutrition-focused intervention such as this one. These roles could be completed by individuals with training in quality improvement employed at the hospitals, or through networks or shared exchanges whereby teams, including dietitians, from different sites support each other. In addition, our resource-intense methods of assessing barriers and tailoring were classified as ‘high’. Given that many of the identified barriers were common across participating sites and that the subsequently selected change strategies were also similar (data not shown) [35], an intervention tailored to these common barriers may be as effective as one that includes the additional steps of a local barriers assessment and tailoring to these site-specific barriers.

The five participating sites were a highly selective subgroup of ICUs (that is, based in North America, minimum eight beds, low performing, presence of a dietitian, predominantly medical patients). Further investigation is required to clarify the optimal tailoring method in ICUs with different characteristics, organizational cultures, and healthcare systems.

## Conclusion

The results of the PERFECTIS study are promising, indicating that a multifaceted, interdisciplinary tailored approach to improving adherence to critical care nutrition guidelines is feasible, and may decrease barriers to enterally feeding critically patients. However, the complexity of this approach may attenuate its application in practice. Potential refinements to the intervention based on the lessons learned from this preliminary study include incorporating common components of the action plans as standard facets of the intervention, a readiness to change assessment at baseline to evaluate ICU ability to manage and accept the proposed changes [36], training for the guideline implementation team on leadership, teamwork, and quality improvement, and a longer implementation period or assessment of change in practice after several time intervals (for example, 12, 18, 24, 36 months). These modified components will ensure a more parsimonious intervention. The proposed changes will need to be piloted prior to proceeding to designing and conducting a large interventional study to formally evaluate the effectiveness of the approach.

## Key messages

• Multiple factors or barriers can impede the provision of adequate EN to critically ill patients.

• The optimal methods for identifying and overcoming barriers is unclear.

• A potential method involves the following five-step process: step 1, nutrition practice audit to determine gaps between guideline recommendations and actual practice; step 2, staff survey to identify barriers to enterally feeding patients; step 3, focus group to prioritize these barriers and brainstorm interventions to overcome the prioritized barriers; step 4, a 12-month implementation phase including bimonthly progress meetings; and step 5, evaluation of the intervention.

• The feasibility of this tailored intervention was demonstrated through a before–after study in seven ICUs in North America.

## Abbreviations

EN: enteral nutrition; IQR: interquartile range; PERFECTIS: PERFormance Enhancement of the Canadian nutrition guidelines by a Tailored Implementation Strategy; PN: parenteral nutrition; RCT: randomized controlled trial.

## Competing interests

The authors declare that they have no competing interests.

## Authors’ contributions

NEC and DKH were responsible for the study conception and design, obtaining the grant to fund this work, and leading the observational site visits. NEC performed the data analysis and was responsible for drafting the manuscript. LM assisted in coordinating the data collection and the site visits. DKH and DC provided methodological and statistical expertise, and helped to interpret the results. All authors made critical revisions to the manuscript. All authors read and approved the final manuscript.

## Supplementary Material

Additional file 1 **Presents the barriers to enterally feeding critically ill patients questionnaire.** Questionnaire distributed to ICU staff to identify local barriers to provision of EN. Click here for file

Additional file 2 Is a table presenting exposure to and nurse ratings of the usefulness of strategies used to implement action plans. Click here for file

Additional file 3 **Is a table presenting barriers score at baseline and follow-up and the change in barriers score for each item, subscale, and overall.** Barriers scores were calculated by awarding 1, 2, or 3 points if the respondent identified an item as a ‘somewhat important’, ‘important’ or ‘very important’ barrier respectively. If an item was rated 1 to 4 (that is, ‘not at all important’ to ‘neither important or unimportant’) it was awarded 0 points. The barriers score was calculated by dividing the awarded points for each item by the maximum potential points (that is, 3 points) and expressed as a percentage. The overall and domain barriers score is the mean score for all the items, and domain items, respectively. Change in barriers score were calculated as the score at baseline subtracted from score at follow-up. SD, standard deviation. Click here for file
