# Supplementary material for: Implementing a multifaceted tailored intervention to improve nutrition adequacy in critically ill patients: results of a multicenter feasibility study
Source: Crit Care. 2014 May 11;18(3):R96. doi: 10.1186/cc13867 (PMC4229943; doi:10.1186/cc13867)
Supplement: Additional file 2 — Is a table presenting exposure to and nurse ratings of the usefulness of strategies used to implement action plans. [file cc13867-S2.pdf]

Additional File 2: Table Describing Exposure to and Nurses Ratings of the Usefulness of Strategies used to Implement the Action Plans

	Exposed		Useful[a]
	n/N (%)	Site range	Median (Site range)
**Bed-side Tools**			
EN Initiation orders and/or bedside algorithm	65/80 (81%)	59-92%	4 (4-5)
Motility agent order	10/13 (77%)	N/A	4 (N/A)
Protocol for withholding feeds	5/6 (83%)	N/A	5 (N/A)
Change volume for interruptions	33/39 (85%)	77-94%	4 (4-4)
Daily monitoring checklist	13/13 (100%)	N/A	4 (N/A)
**Access to Resources**			
Par stock of EN formula	71/81 (88%)	83-91%	4 (4-5)
RD coverage schedule	29/41 (71%)	59-85%	4 (3-5)
**Education/ Information**			
Nutrition section in ICU protocol	16/21 (76%)	N/A	4 (N/A)
Bedside huddles	53/72 (74%)	46-94%	4 (4-4)
Informal education by RD on rounds	35/38 (92%)	86-100%	4 (4-4)
Lunch and learns	4/6 (67%)	N/A	4 (N/A)
Nutrition Information Sheets (NIBBLE)	41/79 (52%)	0-82%	4.5 (3.5-4)
Newsletter	6/12 (50%)	N/A	2.5 (N/A)
Intranet posting	10/21 (48%)	N/A	3.5 (N/A)
Grand Rounds presentation	11/27 (41%)	33-67%	4 (4-4.5)
**Reminder**			
Posters	63/77 (82%)	67-94%	4 (3-4.5)

[a] Rating scale: 1=useless, 2=somewhat useless, 3=neutral, 4=somewhat useful, 5=very useful
N/A – strategy employed at single site only
