# Supplementary material for: Implementing a multifaceted tailored intervention to improve nutrition adequacy in critically ill patients: results of a multicenter feasibility study
Source: Crit Care. 2014 May 11;18(3):R96. doi: 10.1186/cc13867 (PMC4229943; doi:10.1186/cc13867)
Supplement: Additional file 3 — Is a table presenting barriers score at baseline and follow-up and the change in barriers score for each item, subscale, and overall. Barriers scores were calculated by awarding 1, 2, or 3 points if the respondent identified an item as a ‘somewhat important’, ‘important’ or ‘very important’ barrier respectively. If an item was rated 1 to 4 (that is, ‘not at all important’ to ‘neither important or unimportant’) it was awarded 0 points. The barriers score was calculated by dividing the awarded points for each item by the maximum potential points (that is, 3 points) and expressed as a percentage. The overall and domain barriers score is the mean score for all the items, and domain items, respectively. Change in barriers score were calculated as the score at baseline subtracted from score at follow-up. SD, standard deviation. [file cc13867-S3.pdf]

Additional File 3: Table describing the barriers score at baseline and follow up and the change in barriers score for each item, subscale, and overall

	Before (n=182)	After (n=118)		Site Range		
**Barrier**	**Mean Score (SD)**	**Mean Score (SD)**	**Change in Score**	**Min**	**Max**	**P-value**
**Overall Score**	**30.5 (23.1)**	**20.8 (22.7)**	**-9.7**	**-4.3**	**-26.04**	**0.0004**
**Subscale 1: Guideline Recommendations and implementation Strategies**	**23.8 (24.4)**	**16.9 (26.7)**	**-6.9**	**-1.6**	**-18.1**	**0.02**
1. Current scientific evidence supporting some nutrition interventions is inadequate to inform practice.	22.9 (29.9)	17.8 (29.8)	-5.1	0.8	-21.0	0.15
2. The current national guidelines for nutrition are not readily accessible when I want to refer to them.	31.3 (26.3)	15.8 (29.5)	-15.5	-7.2	-25.0	<0.0001
3. The language of the recommendations of the current national guidelines for nutrition are not easy to understand.	21.6 (31.9)	13.8 (27.0)	-7.8	-2.7	-27.4	0.03
4. No feeding protocol in place to guide the initiation and progression of enteral nutrition.	24.5 (31.3)	19.5 (33.6)	-5.1	1.1	-15.1	0.19
5. Current feeding protocol is outdated.	18.5 (29.4)	17.5 (32.2)	-1.0	0.8	-15.7	0.79
**Subscale 2: ICU Resources**	**30.5 (31.1)**	**20.9 (28.7)**	**-9.6**	**0.3**	**34.1**	**0.008**
6. Not enough nursing staff to deliver	18.9 (31.8)	11.9 (27.4)	-7	0.2	-33.3	0.05

adequate nutrition.						
7. Enteral formula not available on the unit.	34.2 (38.3)	22.3 (36.2)	-11.9	-3.8	-30.2	0.007
8. No or not enough feeding pumps on the unit.	38.3 (38.5)	28.5 (36.5)	-9.7	2.3	-38.9	0.03
**Subscale 3: Dietician Support**	**29.7 (26.9)**	**21.1 (28.5)**	**-8.6**	**-1.4**	**-18.1**	**0.009**
9. Waiting for the dietician to assess the patient.	28.2 (33.8)	17.2 (30.7)	-11.0	-5.0	-23.0	0.004
10. Not enough dietician time dedicated to the ICU during regular weekday hours.	22.9 (32.8)	13.8 (27.7)	-9.1	-2.6	-19.8	0.01
11. No or not enough dietician coverage during weekends and holidays.	37.4 (36.2)	28.0 (36.7)	-9.4	-2.4	-19.4	0.03
12. Not enough time dedicated to education and training on how to optimally feed patients.	30.4 (31.8)	25.4 (36.6)	-5.0	2.2	-17.9	0.21
**Subscale 4: Delivery of Enteral Nutrition to the Patient**	**37.4 (27.7)**	**25.4 (27.3)**	**-12.0**	**-2.3**	**-36.0**	**0.0003**
13. Delay in physicians ordering the initiation of EN.	37.9 (34.6)	27.7 (33.3)	-10.2	-2.1	-27.0	0.01
14. No feeding tube in place to start feeding.	34.2 (36.1)	24.0 (36.4)	-10.2	0.8	-46.8	0.02
15. Delays in initiating motility agents in patients not tolerating enteral nutrition (i.e. high gastric residual volumes).	32.8 (34.6)	21.8 (31.8)	-11.0	2.2	-44.0	0.005

16. Delays and difficulties in obtaining small bowel access in patients not tolerating enteral nutrition (i.e. high gastric residual volumes).	40.5 (34.7)	29.9 (35.5)	-10.5	-0.1	-35.7	0.01
17. In resuscitated, hemodynamically stable patients, other aspects of patient care still take priority over nutrition.	41.6 (35.2)	23.7 (32.4)	-17.8	-12.7	-26.6	<0.0001
**Subscale 5: Critical Care Provider Attitudes and Behaviour**	**31.1 (28.0)**	**19.8 (23.6)**	**-11.4**	**-2.6**	**-21.9**	**0.0003**
18. Non-ICU physicians (i.e. surgeons, gastroenterologists) requesting patients not be fed enterally.	35.2 (35.5)	28.8 (35.9)	-6.4	-2.0	-20.3	0.13
19. Nurses failing to progress feeds as per the feeding protocol.	27.1 (33.9)	14.4 (26.3)	-12.7	-0.3	-22.6	0.0003
20. Fear of adverse events due to aggressively feeding patients.	27.5 (33.1)	14.4 (26.7)	-13.1	-8.9	-25.8	0.0002
21. Feeding being held too far in advance of procedures or operating room visits.	34.8 (35.7)	21.4 (31.9)	-13.3	4.1	-23.8	0.0011
